# Risk factors associated with inappropriate empirical antimicrobial treatment in bloodstream infections. A cohort study

**DOI:** 10.3389/fphar.2023.1132530

**Published:** 2023-03-24

**Authors:** Beatriz Dietl, Lucía Boix-Palop, Laura Gisbert, Aina Mateu, Gemma Garreta, Mariona Xercavins, Cristina Badía, María López-Sánchez, Josefa Pérez, Esther Calbo

**Affiliations:** ^1^ Department of Infectious Diseases, Hospital Universitari Mútua de Terrassa, Barcelona, Spain; ^2^ Faculty of Medicine, Infectious Diseases, Universitat Internacional de Catalunya, Barcelona, Spain; ^3^ Department of Clinical Pharmacy, Hospital Universitari Mútua de Terrassa, Barcelona, Spain; ^4^ CatLab, Department of Microbiology, Barcelona, Spain; ^5^ Infection Control Nursing Team, Hospital Universitari Mútua de Terrassa, Barcelona, Spain

**Keywords:** bacteremia, bloodstream infections (BSI), anti-bacterial agents-therapeutic use, risk factors, antimicrobial therapy, antimicrobial stewardship (ASP) intervention

## Abstract

**Introduction:** Bloodstream infections (BSI) are a major cause of mortality all over the world. Inappropriate empirical antimicrobial treatment (i-EAT) impact on mortality has been largely reported. However, information on related factors for the election of i-EAT in the treatment of BSI in adults is lacking. The aim of the study was the identification of risk-factors associated with the use of i-EAT in BSI.

**Methods:** A retrospective, observational cohort study, from a prospective database was conducted in a 400-bed acute-care teaching hospital including all BSI episodes in adult patients between January and December 2018. The main outcome variable was EAT appropriation. Multivariate analysis using logistic regression was performed.

**Results:** 599 BSI episodes were included, 146 (24%) received i-EAT. Male gender, nosocomial and healthcare-associated acquisition of infection, a high Charlson Comorbidity Index (CCI) score and the isolation of multidrug resistant (MDR) microorganisms were more frequent in the i-EAT group. Adequation to local guidelines’ recommendations on EAT resulted in 91% of appropriate empirical antimicrobial treatment (a-EAT). Patients receiving i-EAT presented higher mortality rates at day 14 and 30 when compared to patients with a-EAT (14% vs. 6%, *p* = 0.002 and 22% vs. 9%, *p* < 0.001 respectively). In the multivariate analysis, a CCI score ≥3 (OR 1.90 (95% CI 1.16–3.12) *p =* 0.01) and the isolation of a multidrug resistant (MDR) microorganism (OR 3.79 (95% CI 2.28–6.30), *p <* 0.001) were found as independent risk factors for i-EAT. In contrast, female gender (OR 0.59 (95% CI 0.35–0.98), *p =* 0.04), a correct identification of clinical syndrome prior to antibiotics administration (OR 0.26 (95% CI 0.16–0.44), *p* < 0.001) and adherence to local guidelines (OR 0.22 (95% CI 0.13–0.38), *p <* 0.001) were identified as protective factors against i-EAT.

**Conclusion:** One quarter of BSI episodes received i-EAT. Some of the i-EAT related factors were unmodifiable (male gender, CCI score ≥3 and isolation of a MDR microorganism) but others (incorrect identification of clinical syndrome before starting EAT or the use of local guidelines for EAT) could be addressed to optimize the use of antimicrobials.

## 1 Introduction

Bloodstream infections are one of the main causes of morbidity and mortality worldwide (Weinstein et al., 1997; Wisplinghoff et al., 2004; Rodriguez-Creixems et al., 2008) and even in 2019, septicemia remained the 12th leading cause of mortality in the United States (Xu et al., 2021).

Appropriation of empirical antimicrobial treatment (EAT) has become a difficult decision due to the increase of antimicrobial resistance to antibiotics in the past few years, both in the healthcare and the community (Arias and Murray, 2009). This decision needs to find the adequate balance between using a broad-spectrum antibiotic, which may not be necessary and has a negative impact on the environment, leading to an increase in the emergence of antibiotic-resistant bacteria, and choosing a proper option, which targets the most frequently implied pathogens in the suspected infection (Paterson and Rice, 2003).

Inadequate EAT (i-EAT) has been largely associated with an increased mortality, compared with an antibiotic regimen that is adequately active against the organisms causing the infection. Many studies have focused on assessing the impact on mortality of an inadequate empirical antimicrobial treatment not only in specific scenarios, like the intensive care units (ICU) (Alvarez-Lerma; Ibrahim et al.; Kollef et al.; Luna et al.; Rello et al.; Kreger et al., 1980; Savage et al., 2016) but also it has been analysed in a broad spectrum of infections (Mettler et al., 2007; Paul et al., 2010). Concretely in patients with bacteremia, inadequate therapy has demonstrated to negatively impact on mortality in specific populations like oncohematological patients with febrile neutropenia (Martinez-Nadal et al., 2020; Chumbita et al., 2022) or cirrhotic patients (Park, 2015). Also, results from a very recent meta-analysis indicated that i-EAT substantially had a negative impact on survival of patients with BSIs at short and long term (Hung et al., 2022).

Despite of the relevance of choosing i-EAT, there is much less evidence on the risk factors associated with this practice. Some studies have focused on concrete patient-related factors (McDonald et al., 2005; Retamar et al., 2013) while others have analysed heterogeneous clinical syndromes (Mettler et al., 2007), have small sample size (Bai et al., 2021) or have focused on specific populations like neonates (Hsu et al., 2015) or immunosuppressed patients (Martinez-Nadal et al., 2020; Chumbita et al., 2022), which makes their findings ungeneralizable to general adult population. More recently, some studies have focused on the impact of isolation of multidrug resistant (MDR) strains on the adequation of empirical antimicrobial treatment choice (Girometti et al., 2014; Carrara et al., 2018) showing higher rates of i-EAT when a MDR microorganism was identified and thus, a negative impact on mortality. In a very recent study designed to assess the predictors and mortality risk of discordant EAT for BSI in a large cohort of US hospitals (Kadri et al., 2021) it was also identified the isolation of a MDR microorganism as the main factor associated with receiving i-EAT (*in-vitro* susceptibility-discordant).

Therefore, it is understandable that in the past few years, the efforts of the Antimicrobial Stewardship Programs (ASP) had moved towards the design of EAT local guidelines, considering the possibility of participation of resistant microorganisms (according to their local rates of resistant strains), not only in the healthcare setting, but also in community-acquired infections. The use of these guidelines has been associated with appropriate EAT (a-EAT) and thus with reduced mortality (Enoch et al., 2011).

Hence, despite some studies have pointed out risk factors for inappropriate empirical antimicrobial treatment for bloodstream infections in specific settings, information on factors related to i-EAT is still scarce. To address this, the aim of the present study was to identify the risk factors associated with the use of inappropriate empirical antimicrobial treatment in bloodstream infections. We hypothesised that there might be some potentially modifiable factors (prescription-related factors) that could be addressed to optimize the EAT choice.

## 2 Material and methods

### 2.1 Study design, setting and patients

A retrospective, observational, cohort study carried out in a 400-bed teaching hospital in Spain (Hospital Universitari Mútua de Terrassa, with a mean annual admission of 24,000 patients, catchment area of 350,000 inhabitants).

All consecutive episodes of clinically significant bloodstream infection (BSI) in adults (≥16 years old) have been prospectively recorded since 1989, by the Bloodstream Infection Surveillance Program, a multidisciplinary team which makes a prospective clinical follow-up of all patients with documented bacteremia. All BSI cases identified from this database, between January 2018 and December 2018, were included and retrospectively reviewed by three independent Infectious Diseases consultants. A patient could be included in the study more than once if they have different BSIs at different times. Patients were followed-up for 30 days.

The need for informed consent was waived because this is an observational quality control study without any effect on patients’ medical management. STROBE recommendations were followed to strengthen the reporting of the study results (Supplementary Table S1) (von Elm et al., 2014).

### 2.2 Variables and definitions

Main exposure variable was empirical antimicrobial treatment (EAT) appropriation. The administration of an antimicrobial with *in-vitro* activity (according to susceptibility data) at recommended doses within the first 24 h after the blood cultures had been performed (Savage et al., 2016) was defined as a-EAT. Otherwise, it was defined as i-EAT, including also BSI episodes in which EAT was not prescribed (no-EAT).

Other clinical variables collected included age, gender, comorbidities based on Charlson Comorbidity Index (CCI) (Charlson et al., 1987); severity of the BSI using the Pitt bacteremia score (Hilf et al., 1989); shock according to the usual definitions (Levy et al., 2003); lieu of acquisition; ability to identify the clinical syndrome by prescribing physicians, defined as the concordance between the recorded diagnosis before the initiation of EAT by the prescriber and the final diagnosis at hospital discharge; adherence to recommendations made on local guidelines for EAT; source of BSI, defined according to the CDC criteria (Horan et al., 2008) and aetiology. Outcomes were all-cause mortality at days 14th and 30th.

The acquisition lieu of the infection was recorded based on Friedman et al. definitions (Friedman et al., 2002): a nosocomial BSI was considered when it occurred 48 h after admission or if it developed in a patient discharged from hospital in the previous 14 days; healthcare-associated (HC-A) BSI was diagnosed if the patient fulfilled at least one of the following criteria: i) Resided in a nursing home or long-term care facility 30 days before the episode; ii) Had been hospitalized in an acute care hospital for ≥48 h, 90 days before the episode; iii) Attended a hospital or hemodialysis clinic or received intravenous therapy, 30 days before the episode; and/or iv) Had received intravenous therapy, wound care, enteral nutrition or healthcare at home, 30 days before the episode. Otherwise, the BSI was considered as community acquired.

Local guidelines for EAT have been developed at our institution since 2012–2013 by the Antimicrobial Stewardship Program (ASP). They include 13 different syndromes. Local guidelines’ recommendations suggest active *in-vitro* EAT for every of these syndromes according with the most frequent aetiology in our setting. A clinical syndrome which had already been included in the local guidelines, with a specific EAT recommendation was considered as an Antimicrobial Stewardship Program syndrome (ASP-syndrome).

When the origin of the BSI was uncertain despite of careful examination of the clinical and microbiological data, BSI was considered as unknown origin.

Pathogens were considered multidrug resistant (MDR) if they met resistance criteria according to Magiorakos et al. (Magiorakos et al., 2012) or if they were among the ESKAPE microorganisms previously defined (Boucher et al., 2009).

Data were collected from the medical charts and electronic medical records.

### 2.3 Microbiological studies

The recommendations of the Spanish Society of Infectious Diseases and Clinical Microbiology (SEIMC) were followed for performing, processing, and interpreting the blood cultures (Cisneros-Herreros et al., 2007). Antibiotic susceptibility and extended-spectrum beta-lactamase (ESBL) production were interpreted according to the European Committee on Antimicrobial Susceptibility Testing (EUCAST 2018).

### 2.4 Statistical analysis

All statistical analyses were performed using the STATA RELEASE 14 software (StataCorp LP, College Station, TX, United States). Categorical variables are presented using counts and percentages and continuous variables as means and standard deviation (SD) or medians and interquartile range (IQR). Univariate analysis was performed using the chi-squared or Fisher exact test, and the Student t-test or Mann–Whitney *U*-test for comparison of categorical and continuous variables, respectively. A 2-sided *p* < 0.05 was considered statistically significant.

Multivariate analysis was performed by logistic regression. Starting with all variables that showed a trend towards an association (*p* < 0.2), a best subset regression procedure was used to identify the most suitable and parsimonious multivariate model, i.e., the one with the lowest Akaike information criterion, which is a well-known parameter of the goodness of fit of the model (Akaike, 1992). Differences were considered statistically significant at the two-sided *p* < 0.05 level.

The validity of the models was evaluated using the Hosmer-Lemeshow test for estimating goodness of fit to the data, and its discrimination ability by the area under the Receiver Operating Characteristics (ROC) curve.

## 3 Results

### 3.1 Baseline characteristics and outcomes

A total of 599 BSI episodes were included during the study period, 453 (75.6%) received a-EAT and 146 (24.4%) i-EAT.

Table 1 shows the epidemiological and clinical characteristics at admission and the outcomes of the 599 episodes of BSI, as well as the comparison between BSI episodes receiving a-EAT and i-EAT by univariate analysis.

**TABLE 1:** General characteristics of the BSI episodes, and comparison between a-EAT and i-EAT.

Variables	a-EAT N = 453	i-EAT N = 146	Total N = 599	a-EAT vs. i-EAT *p*-value
Age (y), median (IQR)	73 (62–83)	73 (64–83)	73 (63–83)	0.88
**Gender, male**	**272 (60)**	**105 (71.9)**	377 (62.9)	**0.01**
**Charlson Comorbidity Index score ≥3**	**235 (51.9)**	**92 (63)**	327 (54.6)	**0.02**
Healthcare relation				
- **Community acquired**	**248 (54.8)**	**48 (32.9)**	296 (49.4)	**<0.001**
**- Nosocomial**	**105 (23.2)**	**50 (34.3)**	155 (25.6)	**0.01**
- **Healthcare-associated**	**100 (22.1)**	**48 (32.9)**	148 (24.7)	**0.01**
**Identified clinical syndrome**	**354 (78.3)**	**65 (44.5)**	419 (70.1)	**<0.001**
ASP-syndrome^a^	391 (90.1)	128 (94.8)	519 (91.2)	0.09
EAT according to local guidelines	257 (65.7)	25 (19.7)	282 (54.4)	<0.001
**MDR** ^b^ **microorganism**	**82 (18.1)**	**62 (43.1)**	144 (24.2)	**<0.001**
Pitt score, median (IQR)	0 (0–1)	0 (0–1)	0 (0–1)	0.82
Septic shock	81 (17.8)	21 (14.5)	102 (17.1)	0.34
ICU admission	56 (12.4)	18 (12.3)	74 (12.4)	0.99
**Mortality at day 30**	**43 (9.5)**	**32 (21.9)**	**75 (12.5)**	**<0.001**

y: years.

^a^ ASP-syndrome: Antimicrobial Stewardship Program syndrome included in the local guidelines with a specific empirical antimicrobial treatment recommendation.

^b^ MDR: multidrug resistant.

Variables found statisticallly significant.

Mortality at days 14 and 30 was higher in the i-EAT episodes (14.4% vs. 6.2%, *p =* 0.002 at day 14% and 21.9% vs. 9.5%, *p* < 0.001 at day 30 respectively). There was no association between i-EAT and mortality. Data of univariate and multivariate analysis for mortality at days 14 and 30 are shown in Supplementary Table S2.

### 3.2 Source, aetiology and antimicrobial resistance of bloodstream infections

**FIGURE 1:**
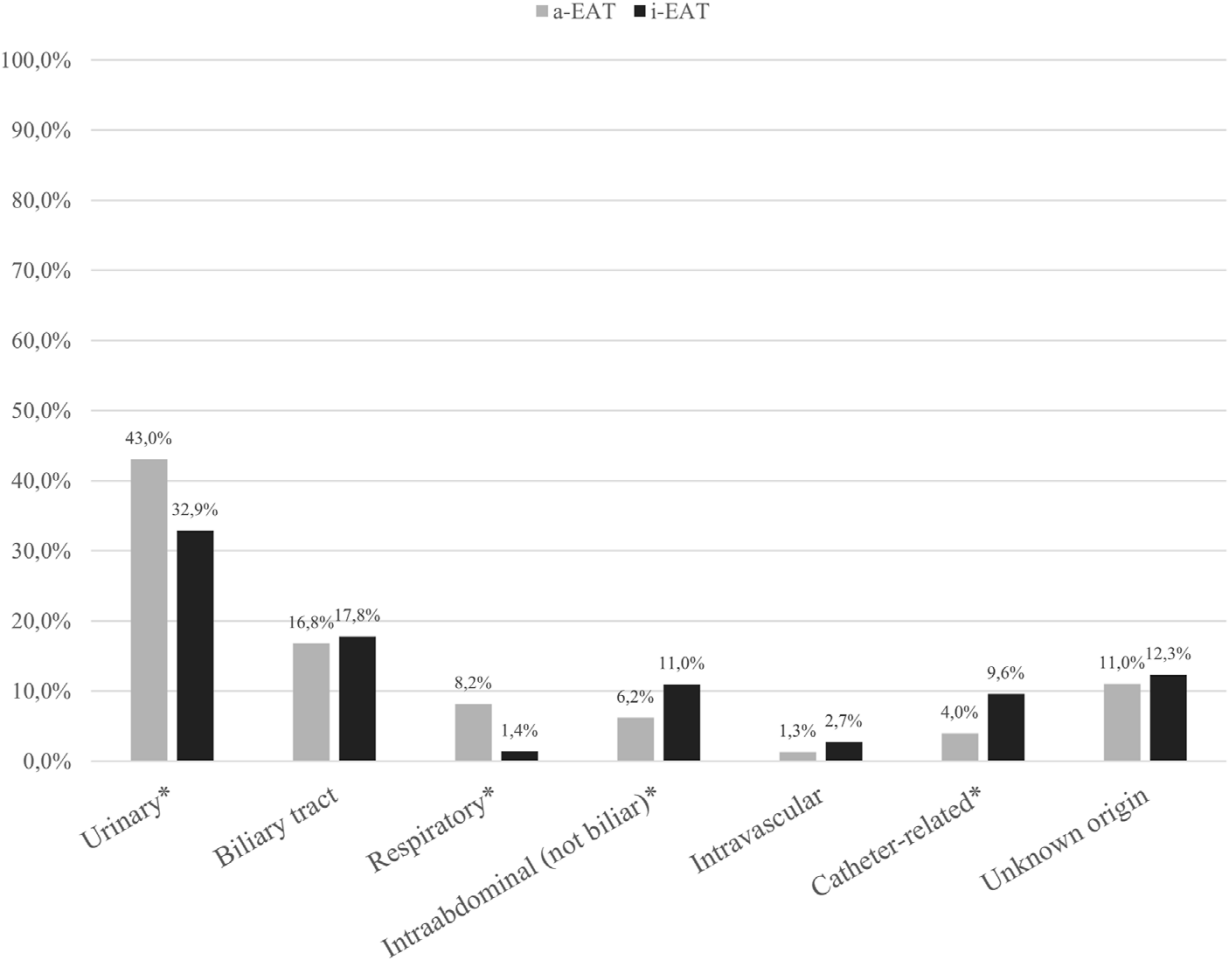
Empirical antimicrobial treatment appropriation according to infection source.

Figure 1 shows EAT appropriation according to the BSI source and Table 2 the most frequently isolated microorganisms. Within the 599 BSI episodes, 35 (5.8%) were polymicrobial. *Escherichia coli* was the most frequently isolated pathogen (47% of all the bacteremia episodes).

**TABLE 2:** Etiology of the BSI episodes and antimicrobial appropriation.

Microorganism	N	a-EAT	i-EAT	*p*
***Escherichia coli***	**241**	**216 (89.6)**	**25 (10.4)**	**<0.001**
**ESBL-producing *E.coli***	**41**	**15 (36.6)**	**26 (63.4)**	**<0.001**
*Klebsiella pneumoniae*	48	39 (81.3)	9 (18.7)	0.34
ESBL-producing *K.pneumoniae*	17	10 (58.8)	7 (41.2)	0.10
**AmpC-producing Gram-negative bacilli**	**39**	**24 (61.5)**	**15 (38.5)**	**0.03**
*Pseudomonas aeruginosa*	13	9 (69.2)	4 (30.8)	0.59
*Staphylococcus aureus*	37	23 (62.2)	14 (37.8)	**0.05**
***MRSA*** ^a^	**3**	**0**	**3 (100)**	**0.002**
**Other *Staphylococcus* spp.**	**17**	**8 (47.1)**	**9 (52.9)**	**0.005**
***S.pneumoniae***	**31**	**30 (96.8)**	**1 (3.2)**	**0.01**
***E.faecalis***	**20**	**11 (55)**	**9 (45)**	**0.03**
***E.faecium***	**21**	**8 (38.1)**	**13 (61.9)**	**<0.001**
Other *Streptococcus* spp.	45	36 (80)	9 (20)	0.48
Other microorganisms	35	28 (80)	7 (20)	0.54
Polymicrobial^b^	35	23 (65.7)	12 (34.3)	0.15

^a^ Methicillin-resistant *Staphylococcus aureus*.

^b^ Polymicrobial infections include some of the previously listed microorganisms (i.e., *Escherichia coli*) so the global sum of antibiotics is higher than the global number of BSI, episodes (N = 599).

Variables found statisticallly significant.

According to the previous definitions, 24.2% of isolated microorganisms were considered MDR. Within the episodes in which a-EAT was administered, 82/453 (18.1%) were caused by a MDR microorganism (vs. 62/146 (43.1%) which received i-EAT, *p* < 0.001).

Mortality rates in BSI episodes caused by a MDR microorganism were 12.5% at day 14% and 17.4% at day 30 with no statistical differences between i-EAT and a-EAT groups (55.6% vs. 44.4%, *p =* 0.25 at day 14% and 52% vs. 48%, *p =* 0.32 at day 30 respectively). Regarding the lieu of acquisition, 32.6% of the BSI episodes caused by a MDR microorganism were community-acquired, 28.5% were nosocomial and 38.9% were HC-A (*p* < 0.001). Differences in the appropriateness of EAT caused by a MDR microorganism according to the healthcare delivery setting were not found (*p =* 0.70).

### 3.3 Adequate identification of clinical syndrome and EAT regimens

In 177 BSI episodes physicians did not identify the clinical syndrome before the antibiotic prescription (misdiagnosis rate of 29.6%).

EAT before microbiologic information was prescribed in 549 BSI episodes (91.7%). Considering BSI episodes with no-EAT (49), 23 (47%) were patients without clinical suspicion of infection; in 18 (36.7%) a delayed-initiating antibiotic strategy was chosen due to the clinical stability of the patients and, in the remaining 8 (16.3%), antibiotics were not prescribed because of limitation of therapeutic effort. When the 26 BSI episodes with no-EAT due to clinical decisions (clinical stability of the patient or limitation of therapeutic effort) were excluded, the i-EAT rate decreased to 20.9%. Excluding all the BSI episodes with no-EAT, the i-EAT rate was 17.5%.

Among all the episodes, 519 (91.2%) were considered ASP-syndromes. Antibiotics according to local guidelines’ recommendations were prescribed in 282 out of 519 ASP-syndromes (54.3%); among these, 25 (8.9%) received i-EAT. Eleven of these patients (44%) had a BSI caused by a MDR organism but with no identifiable risk factors for a MDR microorganisms.

Regarding BSI episodes in which the clinical syndrome had been correctly identified but local guidelines’ recommendations were not followed, in 64 out of 127 episodes (50.3%) patients received unnecessary broad-spectrum antibiotics.

The most frequently used antimicrobials for EAT are shown in Table 3. Monotherapy regimens were used in 63% of BSI episodes. Combination therapy was more frequent in the a-EAT group (86% vs. 14%, *p* < 0.001).

**TABLE 3:** Most frequent antibiotics used for empirical therapy.

Antibiotic	N	(%)
Ceftriaxone	166	27.7
Piperacillin/tazobactam	77	12.9
Carbapenems (ertapenem, imipenem or meropenem)	82	13.7
Amoxicillin/clavulanic	62	10.4
Amikacin	56	9.4
Cefotaxime	55	9.2
Ceftazidime	34	5.7
Glucopeptydes/lipopeptydes (vancomycin or daptomycin)	28	4.7
Linezolid	16	2.7
Combination therapy^a^	219	36.6

^a^ Combination therapies include some of the previously listed antibiotics (i.e., ceftriaxone or amoxicillin/clavunate) so the global sum of antibiotics is higher than the global number of BSI, episodes (N = 599).

The unit of admission for the BSI episodes is described in Table 4.

**TABLE 4:** Admission Unit of the BSI episodes and comparison between the a-EAT and i-EAT episodes in relation with the global antimicrobial appropriation.

Admission unit N)	a-EAT (N = 453) (%)	i-EAT (N = 146) (%)	*p*
Emergency Department (156)	110 (24.3)	46 (31.5)	0.08
Internal Medicine (149)	116 (25.6)	33 (22.6)	0.47
Hematology (33)	24 (5.3)	9 (6.2)	0.69
Oncology (27)	19 (4.2)	8 (5.5)	0.52
Gastroenterology (25)	17 (3.8)	8 (5.5)	0.36
Other medical wards^a^ (42)	38 (8.4)	4 (2.7)	0.002
General Surgery (69)	54 (11.9)	13 (8.9)	0.32
Urology (24)	18 (4)	6 (4.1)	0.94
Other surgical wards^b^ (29)	25 (5.5)	4 (2.7)	0.27
Intensive Care Unit (47)	32 (7.1)	15 (10.3)	0.21

^a^ Medical wards: Pneumology, Cardiology, short stay unit.

^b^ Surgical wards: Vascular surgery, Traumatology, Neurosurgery, Cardiology, Ginecology, Otorhinolaryngology.

### 3.4 Risk factors associated with EAT appropriateness

The multivariable logistic regression model used to assess variables associated with i-EAT is shown in Table 5. Male gender, a high CCI score (≥3 points), inadequate identification of clinical syndrome before initiating EAT, not prescribing EAT according to local guidelines and the isolation of MDR microorganisms were identified as risk factors for inappropriate choice of antimicrobial empirical therapy in BSI. The risk factors associated with i-EAT excluding the BSI episodes with no-EAT due to clinical decisions have been also analysed. Data are shown in Supplementary Table S2.

**TABLE 5:** Multivariate analysis.

Variable	OR	CI (95%)	*p*
**Gender, female**	**0.59**	**0.35–0.98**	**0.04**
**Charlson Comorbidity Index score ≥3**	**1.70**	**1.04–2.80**	**0.04**
**Correct identification of clinical syndrome**	**0.23**	**0.13–0.38**	**<0.001**
**EAT according to local guidelines**	**0.22**	**0.13–0.38**	**<0.001**
**Resistant microorganism**	**3.67**	**2.20–6.15**	**<0.001**

*p < 0.05.

Variables found statisticallly significant.

## 4 Discussion

In this cohort study of 599 BSI in adults, EAT was inappropriate in 24.2% cases. We identified that male gender, host comorbidities, incorrect identification of clinical syndrome, lack of adherence to local guidelines and the isolation of MDR microorganisms were independent risk factors for i-EAT.

The i-EAT rate in our study (24.2%) was similar to previous studies (McDonald et al., 2005; Mettler et al., 2007; Retamar et al., 2012; Bai et al., 2021; Kadri et al., 2021) and even smaller than the pooled estimate of 32% in a systematic review of studies on i-EAT (Carrara et al., 2018). The differences between these rates among the studies are probably related to, on one hand, the heterogenicity of the definitions for appropriate empirical antimicrobial treatment and on the other, the variability of evaluated infections and the different rates of resistant microorganisms in every setting. Besides, there are some important differences in the design as well: i.e., in contrast to Kadri’s study, we did not exclude from the appropriateness analysis patients who had not received EAT on the day blood samples were drawn.

Evidence on the risk factors for i-EAT in adult patients with BSI is scarce. Previous studies have tried to point them out, but they either limited the analysis to the impact of MDR (Girometti et al., 2014; Kadri et al., 2021), were small studies (Bai et al., 2021) or identified some predictors to receive i-EAT even though the study was not designed for this purpose (Retamar et al., 2013). Regarding risk factors for i-EAT identified in our study, they could be classified in patient-related (and, therefore, unmodifiable risk factors) and modifiable risk factors.

Gender differences in the prescription of antibiotics had been previously reported (Norris et al., 2005). In our cohort, female gender was a protective factor for receiving a-EAT (OR 0.59 (95% CI 0.35–0.98), *p =* 0.04) as it was in the study conducted by Mettler et al. (Mettler et al., 2007). As the investigators in this study, we also find difficult to explain why women are more suitable to receive a-EAT in a BSI episode. On the other hand, we found that a CCI score of ≥3 was an independent risk factor for i-EAT with an estimated OR 1.90. Cancer (which scores 6 points in the CCI itself) has already been identified as a risk factor for i-EAT (Retamar et al., 2013). Some comorbidities included in the CCI have been identified as risk factors for certain types of pathogens or mechanisms of resistance (Rodríguez-Baño et al., 2010), so they should be taken into account when considering empirical therapy. However, the most important patient-related risk factor identified in our study was the isolation of a MDR microorganism. We found that 24% of all the episodes were due to a MDR microorganism, which is a much lower incidence of MDR than previously reported in recent studies (with variable rates of MDR isolation between 19% and 65%) (Girometti et al., 2014; Kadri et al., 2021). In our study, we found high rates of i-EAT in BSI caused by MDR strains (43%), which was consistent with the reported rates by Girometti (varying between 23% in ESBL-producing *K. pneumoniae* strains and 77% in KPC-producing strains) and Kadri (49%) and identified that a BSI episode caused by a MDR has nearly a 4-fold greater probability of receiving i-EAT. Prevalence of MDR was also identified as the main unmodifiable risk factor associated with i-EAT in Kadri’s study (OR 9.09, 95% CI 7.68–10.76). Moreover, in a 2018 meta-analysis, isolation of MDR pathogens was independently associated with i-EAT (OR 1.11 95% CI 1.07–1.15) (Carrara et al., 2018) but also with higher mortality rates. This scenario deserves some reflections: on one hand, it is essential to consider the potential participation of MDR microorganisms to design active EATs, which is especially relevant in clinical syndromes associated with high mortality. On the other, the use of broad-spectrum antibiotics has been associated with i-EAT itself (Mettler et al., 2007) but, most important, it can have a negative impact on the environment and lead to the emergence of more resistant, difficult-to-treat microorganisms (Paterson and Rice, 2003) for which active drugs are not always available, forcing the use of “second-line” drugs which are less effective and can cause more adverse effects (Rodriguez-Bano et al., 2018; Paul et al., 2022). Hence the importance of avoiding the use of broad spectra antibiotics in clinical syndromes with low mortality risk (i.e., uncomplicated urinary tract infections).

Interestingly, we found some important modifiable factors related to i-EAT in BSI episodes. First, a right clinical diagnosis of the site of infection before antibiotics administration is a protective factor against i-EAT (OR 0.23, (95% CI 0.13–0.38), *p* < 0.001). Recently, the impact of misdiagnoses of infection site previous to antibiotic administration has been analysed (Dregmans et al., 2022). This study identified a misdiagnoses rate of 11.6%, although investigators could not find association between misdiagnoses and worse clinical outcomes as previous studies did (Abe et al., 2019; Hautz et al., 2019; Hussain et al., 2019). Investigators speculated that these controversial results could have been influenced because most patients with misdiagnoses received a broad-spectrum antimicrobial treatment, which was effective in different sites of infection, despite the correct site was not identified. Thus, they also contemplate the possibility that the effects of incorrect diagnosis of the infection site on clinical outcomes may be much wider, and not only limited to the impact on mortality. Some of these related-to-misdiagnosis effects could be (a) The unnecessary use of antibiotics (in our cohort 18% of misdiagnoses received unnecessary broad-spectrum antibiotics, like in Dregmans’ study, where 20% of misdiagnosed patients received unnecessarily antibiotics) or (b) The choice of i-EAT in serious infections like bacteremia which had been related to poor prognosis (Ortega et al., 2007; Paul et al., 2010; Retamar et al., 2012; Carrara et al., 2018), despite we could not find this association in our cohort. Regarding this absence of association between i-EAT and mortality, we contemplate that it could probably been explained by different reasons: 1) The global i-EAT in our cohort was smaller than previous studies (Carrara et al., 2018; Kadri et al., 2021) so the vast majority of the patients received a-EAT which could impact on the global mortality rate; 2) Our participation rate of MDR microorganisms was relatively low (24%) compared to previous studies, where resistance had a significant impact on mortality (Girometti et al., 2014) and 3) Patients from our cohort had low severe infections (septic shock rate 17%, ICU admission rate 12% and a median Pitt score of 0). In 2010 and 2022’s meta-analysis, i-EAT had higher impact on mortality among patients with septic shock at infection onset (Paul et al., 2010; Hung et al., 2022). Thus, in our cohort with mild BSI episodes, this effect could have been lessened.

Second, the lack of adherence to local guidelines for EAT is the other main risk factor identified for i-EAT in our study. We observed a non-adherence to local guidelines’ recommendations rate of 45.6%. In a recent study, adherence to local guidelines was evaluated by annual Point Prevalence Surveys. When prescriptions were incorrect according to local guidelines, after the evaluation of many different aspects (dosing, route of administration, duration, etc.), they were considered as inadequate (Nunez-Nunez et al., 2022). Result from PPS showed a 49.2% of inadequate prescriptions’ rate, similar to the findings in our cohort. In our study, adherence to local guidelines resulted in a-EAT in 91% of episodes (which was a protective factor for i-EAT, with a OR 0.22 (95% CI 0.13–0.38), *p <* 0.001). This finding agrees with a recent study, with a smaller sample size, but with similar design (Bai et al., 2021), which reported that adherence to guidelines produced an a-EAT in 72% of cases in their population. The importance of ASP has been largely studied underlying their beneficial impact on clinical outcomes (better use of antimicrobials, reduction of infections caused by MDR microorganisms and mortality rates, etc.) and also on economic outcomes (Nathwani et al., 2019). There are many types of interventions available for optimizing the use of antimicrobials (Mendelson et al., 2020). Although educational measures could have a modest impact on antimicrobial stewardship, they are usually necessary. First, we found that the adherence to local guidelines can be a protective factor against i-EAT choice. In the study conducted by Kadri et al., investigators suggested that some unmeasured factors could have led antibiotic choices, such as the absence of ASP or the unavailability of appropriate protocols or local guidelines on EAT. Our findings reinforce the essential role of ASP teams. It is indispensable to assure that EAT local guidelines are constantly adapted to, on one hand, the local epidemiology data and, on the other, to patients’ associated factors with the participation of certain microorganisms, to guarantee a proper choice of EAT by physicians who may not be such familiarized with the antimicrobial use. Second, another important protective factor is the correct identification of clinical syndrome before antibiotics prescription. Educational audits or systematic advice should be a cornerstone of the ASP, offered to the clinicians who may be at the front-line of the diagnosis process in order to improve their diagnostic abilities and drive them to a better choice of EAT. All these strategies are focused on improving the appropriateness of EAT which can in turn improve clinical outcomes, considering that i-EAT in bloodstream infections has been largely related with higher mortality (Ortega et al., 2007; Paul et al., 2010; Retamar et al., 2012; Carrara et al., 2018; Hung et al., 2022). The identification of these modifiable risk factors in our study offers many opportunities for improvement and emphasize the importance of the ASP teams.

In 2005 McDonald et al. assessed the specific relationship between HC-A status and ineffective initial therapy (McDonald et al., 2005). Unlike this study, where HC-A was identified as an independent risk factor for i-EAT, we did not find this association. Nevertheless, patients who received i-EAT were more likely to have a nosocomial or HC-A BSI episodes, compared to the community-acquired.

The most frequent sources of BSI were urinary and intraabdominal infections. Urinary and respiratory tract infections received more frequently a-EAT while intraabdominal infections and catheter-related were more frequent sources among i-EAT episodes. *Escherichia coli* was the most frequently isolated pathogen, which is consistent with the most frequent sources of infection where *E. coli* plays a major role.

Our study has many strengths: 1) It provides a relevant sample size and identifies modifiable related factors for i-EAT in BSI episodes, which leads to opportunities for improvement in the ASP strategies. Other previous studies had smaller sample sizes or have focused on concrete, unmodifiable factors (McDonald et al., 2005; Retamar et al., 2013; Girometti et al., 2014; Bai et al., 2021; Kadri et al., 2021). 2) All consecutive adult BSI episodes have been prospectively recorded, and followed for routine clinical practice, so there is no selection bias and there is no patient-loss for the analysis. 3) Local guidelines for EAT have been established in our centre for many years and are well known by all the physicians who make antibiotic prescriptions, so it has been possible to perform a deep analysis on the adherence to the guidelines and the appropriateness of the recommendations. Still, it has some limitations: it is a retrospective, observational study, so conclusions may be interpretated carefully, since more studies need to be performed to confirm the results. Also, it is a single-centre study, performed in a hospital with a consolidated ASP team and low prevalence of MDR microorganisms so the study findings could impact differently in other locations and should not be extrapolated to other institutions without careful assessment of local environment. Finally, we only assessed mortality, but we did not measure other outcomes, like the length of hospital stay or the impact of unnecessary antimicrobial regimens, considering that the effect of i-EAT may be broader (Bai et al., 2021).

In conclusion, one quarter of this cohort of BSI episodes in adults received i-EAT. Some related factors for the choice of i-EAT for bacteremia were unmodifiable, as male gender, high comorbidity or the isolation of a MDR microorganism. However, some important factors were positively associated with a-EAT, as the correct identification of the clinical syndrome before starting EAT and the adherence to local guidelines for EAT, which generate a-EAT in 91% of BSI episodes. The good news is that these modifiable factors could be addressed by the ASP teams to optimize the use of antimicrobials.

## Data Availability

The original contributions presented in the study are included in the article/Supplementary Material, further inquiries can be directed to the corresponding authors.
